# Identification of a novel frameshift mutation in the *ILDR1* gene in a UAE family, mutations review and phenotype genotype correlation

**DOI:** 10.1371/journal.pone.0185281

**Published:** 2017-09-25

**Authors:** Abdelaziz Tlili, Abdullah Fahd Al Mutery, Mona Mahfood, Walaa Kamal Eddine Ahmad Mohamed, Khalid Bajou

**Affiliations:** 1 Department of Applied Biology, College of Sciences, University of Sharjah, Sharjah, United Arab Emirates; 2 Human Genetics and Stem cell laboratory, Research Institute of Sciences and Engineering, University of Sharjah, Sharjah, United Arab Emirates; Central South University Third Xiangya Hospital, CHINA

## Abstract

Autosomal recessive non-syndromic hearing loss is one of the most common monogenic diseases. It is characterized by high allelic and locus heterogeneities that make a precise diagnosis difficult. In this study, whole-exome sequencing was performed for an affected patient allowing us to identify a new frameshift mutation (c.804delG) in the Immunoglobulin-Like Domain containing Receptor-1 (*ILDR1)* gene. Direct Sanger sequencing and segregation analysis were performed for the family pedigree. The mutation was homozygous in all affected siblings but heterozygous in the normal consanguineous parents. The present study reports a first *ILDR1* gene mutation in the UAE population and confirms that the whole-exome sequencing approach is a robust tool for the diagnosis of monogenic diseases with high levels of allelic and locus heterogeneity. In addition, by reviewing all reported *ILDR1* mutations, we attempt to establish a genotype phenotype correlation to explain the phenotypic variability observed at low frequencies.

## Introduction

Deafness is one of the most common sensorineural disorders, affecting one in 1000 individuals. Most congenital cases of deafness have a genetic etiology, and nonsyndromic hearing loss (NSHL) accounts for approximately 80% of genetic deafness [1]. To date, a total of more than 100 NSHL genes have been identified (http://hereditaryhearingloss.org), and most mutations in these genes are inherited in an autosomal recessive pattern. Both allelic and genetic heterogeneities of NSHL make conventional methods (e.g., Sanger sequencing) expensive and time consuming [2]. The development of an efficient and cost-effective approach, whole-exome sequencing (WES), has successfully helped researchers identify new mutations and genes responsible for NSHL [2–7].

The immunoglobulin-like domain containing receptor 1, a predicted type 1 transmembrane protein with a crucial role in the epithelial barrier function in the ear, is encoded by the *ILDR1* gene [8,9]. In 2011, Borck et al., [10] reported eight different homozygous *ILDR1* mutations in affected individuals from 11 unrelated families. Further studies confirmed the implication of this gene in NSHL [2,11,12].

In the present study, we identify a new homozygous frameshift mutation in *ILDR1* in a UAE consanguineous family with autosomal recessive non-syndromic hearing loss (ARNSHL). The mutation p.Glu269ArgfsTer4 in this family is caused by c.804delG. Further, we screened a cohort of 50 UAE familial and sporadic individuals with hearing loss and found that this mutation is unique to this family. To the best of our knowledge, this is the first *ILDR1* identified mutation causing hearing loss in a deaf family from UAE.

## Materials and methods

### Patients

**Fig 1:**
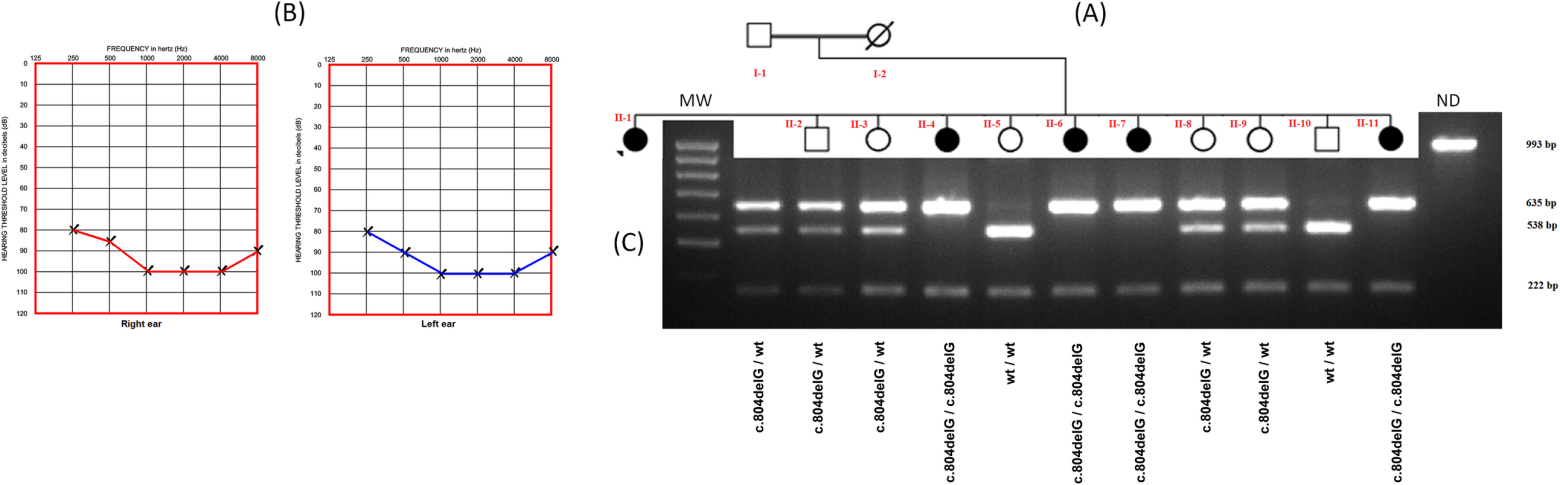
Pedigree of the affected family, Audiogram and PCR-RFLP analysis. (A) Pedigree of the affected family with nonsyndromic hearing loss. Arrow denotes the proband. (B) Audiogram of the proband individual II-1 exhibiting bilateral, severe to profound sensorineural hearing loss. (C) Results of PCR-RFLP analysis of DNA of the affected family with nonsyndromic hearing loss. A 993 bp PCR fragment is digested with FauI restriction enzyme. The wildtype DNA is cleaved into four fragments 538, 222, 136 and 97 bp, whereas the c.804delG mutant allele is cleaved into three fragments 635, 222 and 136 bp in length. MW: DNA Ladder (100bp DNA Ladder, REF G2101) (Promega, USA); ND: undigested PCR product.

In this study, we investigated a UAE consanguineous family transmitting an autosomal recessive non-syndromic severe to profound sensorineural hearing loss (Fig 1). Informed consent was obtained from all participants and from parents of subjects younger than 18 years of age. Clinical history interviews and physical examinations of the family members ruled out the implication of environmental factors for causing the NSHL. Saliva samples were obtained from 12 family members, including 5 hearing-impaired individuals. Saliva samples were also collected from 50 unrelated patients (26 sporadic and 24 familial cases) who were admitted to the UAE deaf association as well as 120 normal individuals as controls. The privacy and anonymity of all participants were protected and only codes were used to label DNA samples. Genomic DNA was extracted from saliva samples using Oragene-DNA (OG-500) Kit (DNA Genotek, CANADA) dx.doi.org/10.17504/protocols.io.jhtcj6n. Written informed consents from all patients or their parents were obtained following audiological and clinical evaluations. The experimental procedures were approved by the Ethics Committee from the University of Sharjah (Sharjah, UAE).

### Whole-exome sequencing and bioinformatics analysis

Sequencing library construction, exome capture, sequencing, and standard data analyses for the affected children in this family was performed by Sengenics. Exome capturing and enrichment was carried out using SureSelect All Exon V5 kit (Agilent Technologies, Santa Clara, CA, USA) following the manufacturers' protocols. Whole exome sequencing was carried out on Illumina HiSeq 2500 system (Illumina, San Diego, CA, USA). Paired end (2×100 bases) DNA sequence reads that passed the quality control i.e phred score > 20 were mapped to the human reference genome build hg19/GRCh37 using the BWA [13] and SAM tools [14] was used for processing BAM files. Genome analysis tool kit (GATK) v2.7.2) [15] was used for calling variants from BAM files. Variants were annotated with gene, existing variations, consequences from dbSNP (build 137), SIFT v5.0.2 [16] and polyphen v2.2.2 [17] using Ensembl Variant Effect Predictor v73 (VEP) [18]. Known variants were annotated by dbSNP and unannotated variants with serious predicted consequences were identified based on SIFT and polyphen which were considered as novel variants. Variants were filtered for increased accuracy using following steps: a) variants were filtered at the read depth (DP) > = 10 b) Variants with >10% i.e > 0.1 minor allele frequency based on 1000 Genome project ((http://www.1000genomes.org/data) dx.doi.org/10.17504/protocols.io.jhscj6e.

### Sanger sequencing

Sanger sequencing was performed on available samples from all affected family members to determine whether the potential mutation in the causative gene co-segregated with the disease phenotype. In order to amplify exon 7 of the *ILDR1* gene, we designed the following primers: ILDR1-7F: TTGATGTCCTGATTCTGAGG and ILDR1-7R: CTCTGTGGTGGAATGAGAGG. The amplified products were then purified using Wizard SV Gel and PCR Clean-up system (Promega, USA) and were consequently sequenced using BigDye Terminator v3.1 Cycle Sequencing Kit (Applied Biosystems, Thermo Fisher Scientific, USA). The resulting sequencing reactions were then purified and precipitated using Ethanol/EDTA/Sodium acetate precipitation method. Capillary sequencing was performed in a Genetic Analyzer 3500 (Applied Biosystems, Thermo Fisher Scientific, USA) and the data were analyzed using Sequencing Analysis software. The sequences were aligned with the published sequence of the *ILDR1* gene (NM_001199799.1) dx.doi.org/10.17504/protocols.io.jhvcj66.

### c.804delG mutation screening

The novel c.804delG mutation, occurring in the seventh exon of the *ILDR1* gene, abolishes a FauI restriction site. The FauI restriction pattern of the exon7 fragment (1003 bp) was used to screen 50 deaf individuals and 120 unrelated healthy UAE individuals. Digestion of PCR products was performed according to manufacturer’s instructions (New England Biolabs, USA), followed by separation on 2% agarose gels dx.doi.org/10.17504/protocols.io.jhwcj7e.

### In silico analysis of DNA variants

In order to determine the consequence of *ILDR1* reported mutations on splicing and translation initiation, we used Human Splicing Finder (version 3.0) (http://www.umd.be/HSF3/) and ORF finder (http://www.geneinfinity.org/sms/sms_orffinder.html) respectively dx.doi.org/10.17504/protocols.io.jhxcj7n.

## Results

### Molecular analysis

**Fig 2:**
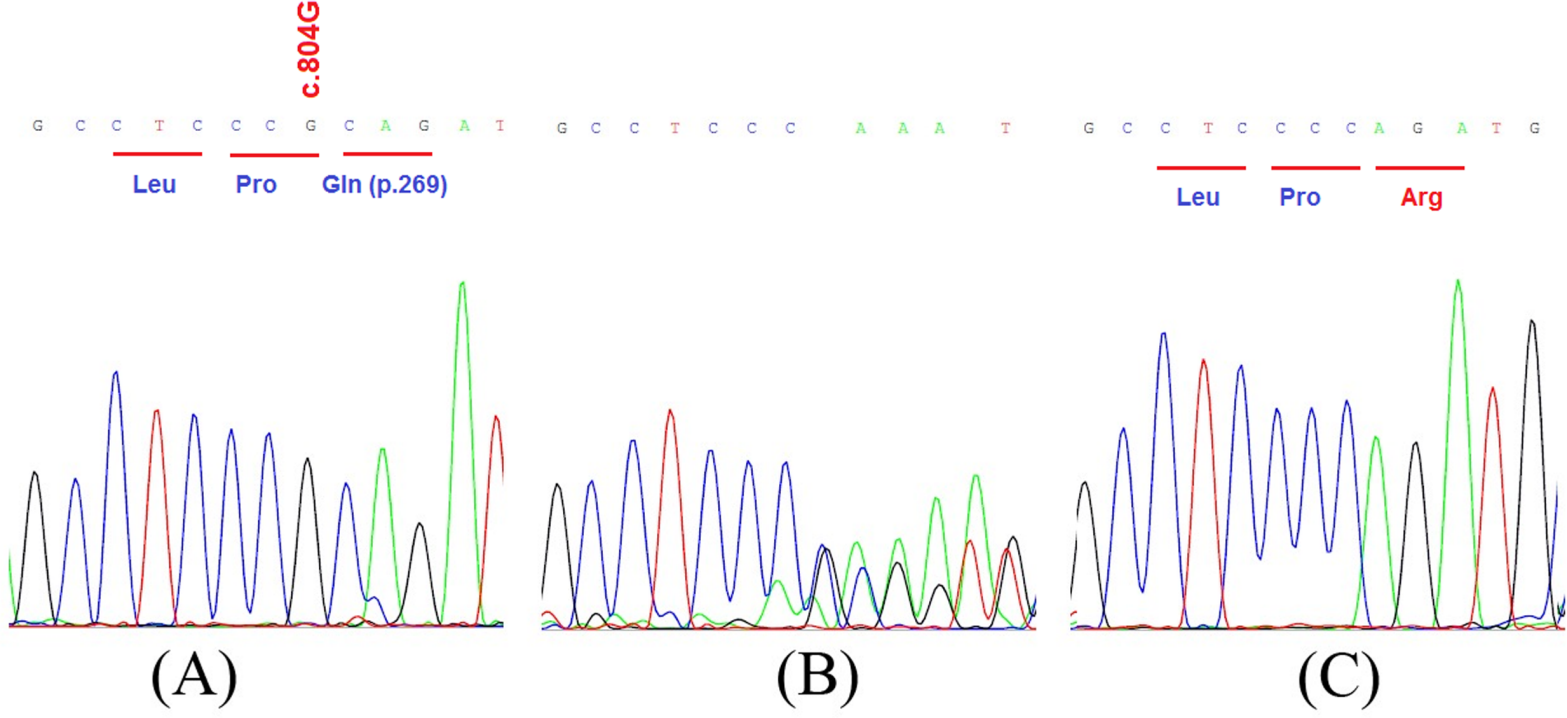
Electropherograms. Homozygous normal individual (A), Heterozygous individual (B), and affected individual with the c.804delG pathogenic variant in the *ILDR1* gene (NM_001199799.1) (C).

A large UAE consanguineous family including five patients with congenital deafness, was investigated (Fig 1A). Clinical examination of all affected individuals ruled out any association with other symptoms and showed no conductive abnormality S1 Table. Hence, we could qualify the hearing loss in the corresponding family as a nonsyndromic autosomal recessive hearing loss. As a routine screening in our laboratory, we first sequenced the *GJB2* gene (Tlili et al., 2017, under revision), the most common gene in ARNSHL, in affected individuals and found no mutations (data not shown). Thus, we performed a WES analysis for the DNA of individual II-1 DNA (Fig 1A). A total of 123484 DNA variations were identified with 6393 missense, 186 frameshift and 64 nonsense variants. This data has been further filtered as follows: (i) only homozygous variants were considered as the disease is recessive and the family was consanguineous, (ii) common variants found in the UAE population and available in our internal database were removed, (iii) variants described in dbSNP (https://www.ncbi.nlm.nih.gov/projects/SNP/) or Exac Browser (http://exac.broadinstitute.org/) with a frequency higher than 0.05% were excluded. After this filtering, only 70 non described DNA variations were kept, 67 among them were in noncoding regions, one silent variant in *HEG1* gene (c.51C>T, p.Leu17Leu), one insertional variation c.308_309insCTG (p.Ala103_Val104insTrp) in the *HLA-DRB5* and one nucleotide deletion c.804delG (p.Glu269ArgfsTer4) in the *ILDR1* gene. These three variants have been analyzed by Mutation Taster algorithm in order to predict their functional impact. Using this analysis, we found that *HEG1* and *HLA-DRB5* variations are predicted to be polymorphisms and only the *ILDR1* variation is predicted to be a "disease causing" variant. To validate this finding and the WES results, we sequenced exon 7 of the *ILDR1* gene in the proband individual and her family members (Fig 2). Our analysis revealed the cosegregation of the c.804delG variant with the hearing loss observed in our studied family. This cosegregation has been also confirmed by PCR-RFLP using FauI restriction enzyme (Fig 1C). In fact, all affected individuals were homozygous for this deletion, both parents were heterozygous and unaffected siblings were either heterozygous or normal homozygous for the wildtype allele. Furthermore, using this restriction enzyme, we demonstrated that this DNA variation was not present in 50 unrelated deaf individuals and 120 normal hearing UAE control individuals. Together, all these analyses and results suggest that the c.804delG deletion within the *ILDR1* gene represents the pathogenic mutation responsible for the ARNSHL observed in this UAE family.

### In silico analysis

**Fig 3:**
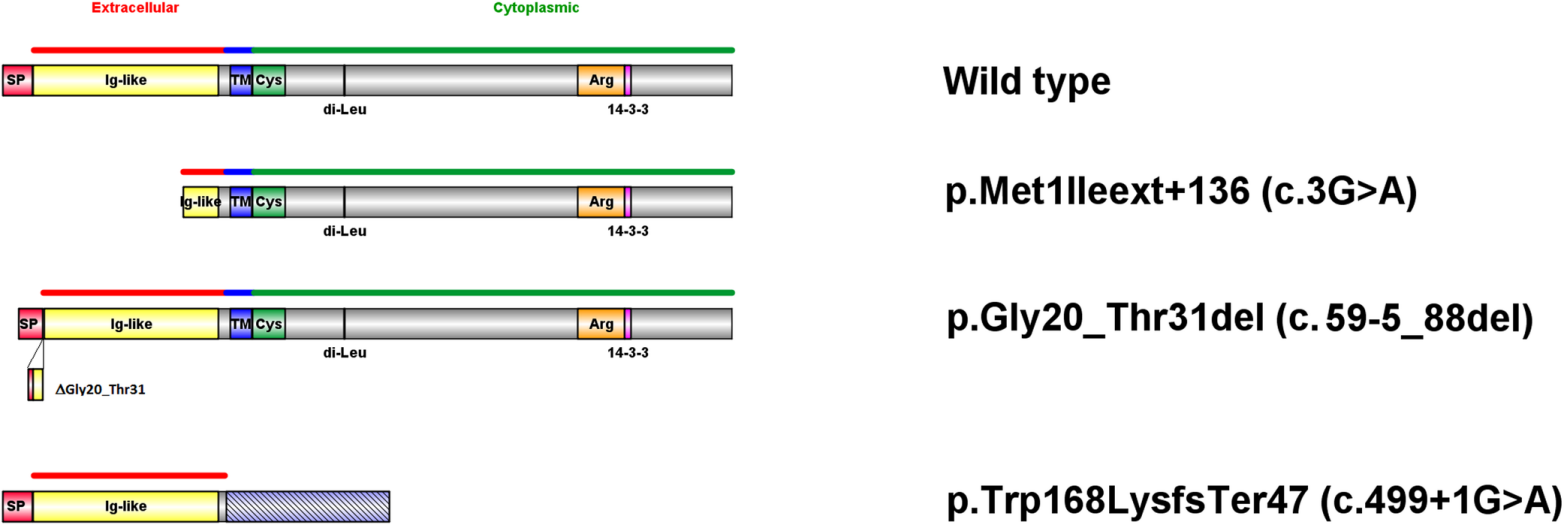
In silico analysis and predicted effect at the protein level of c.3G>A, c.59-5_88del and c.499+1G>A mutations.

In order to establish a genotype phenotype correlation, we reviewed all reported mutations within the *ILDR1* gene and performed an insilico analysis. The study of c.3G>A (p.Met1?) by ORF finder (Table 1, Fig 3) revealed that in the presence of A at position c.3, the next potential initiation codon that can be used is located at position 136 downstream the original one (p.Met136). This alternative translation produces an *ILDR1* protein lacking the signal peptide (SP) and 78% of the Ig domain, however the remaining regions are identical to the wildtype form as the use of p.Met136 doesn't affect the normal frame. The analysis of the c.59-5_88del mutation using HSF (Table 1, Fig 3), revealed a new potential splice acceptor site "cttggcacacagAA" in frame with a consensus value of 84.34 (the value of the original one "tcttggctttagGG" abolished with this mutation, is 84.05). Taking into consideration this new acceptor site, the predicted consequence of c.59-5_88 del mutation on the protein will be an in-frame deletion of 4 aa of the SP and 8 aa of the Ig domain (p.Gly20_Thr31del). Concerning the intronic variation c.499+1G>A, our HSF analysis revealed that the wildtype donor site "TACgtaagt" with a consensus value of 87.01 has been lost and probably the totality of intron 4 (290 bp) will be included in the coding region. This will probably produce a mutant *ILDR1* protein (p.Trp168LysfsTer47) of 214 aa only (Table 1, Fig 3). For the remaining 14 reported mutations, none of them creates a new potential initiation codon, however a few only create new splice sites but their score values were less than the normal ones (data not shown).

**Table 1:** *ILDR1* reported mutations, described effects and predicted consequences using insilico analysis.

DNA Mutation	Described protein mutation	Reference	Predicted insilico protein mutation (this study)
c.3G>A	Use of the next downstream ATG out-of-frame, potentially producing a 43 amino acid polypeptide with no similarity to ILDR1.	[10]	p.Met1Ile*ext*+136
c.59-5_88del	N/D	[10]	p.Gly20_Thr31del
c.82delG	p.V28SfsX31	[3]	-
c.206C>A	p.Pro69His	[19]	-
c.290 G>A	p.Arg97Gln	[10]	-
c.305T>A	p.Val102Glu	[12]	-
c.325_333dupAATGAGCCC	p.Asn109_Pro111dup	[11]	-
c.411delG	p.Trp137CysfsX25	[10]	-
c.499+1G>A	N/D	[10]	p.Trp168LysfsTer47
c.583C>T	p.Gln195X	[10]	-
c.804del G	p.Glu269ArgfsTer4	Present study	-
c.820C>T	p.Q274X	[2]	-
c.942C>A	p.C314X	[3]	-
c.1032delG	p.Thr345ProfsX20	[10]	-
c.1135G>T	p.Glu379X	[10]	-
c.1180delG	p.Glu394SerfsX15	[10]	-
c.1217-1218delTC	p.S406X	[12]	-
c.1358G>A	p.Arg453Gln	[10]	-

N/D: not determined

## Discussion

We report a UAE consanguineous family affected with ARNSHL. The affected members have severe to profound congenital hearing loss. Further clinical examinations ruled out the association with any other symptom, and suggested a nonsyndromic form of hearing loss in the studied family. We first analyzed the affected individual II-1 for mutations in the *GJB2* gene, the most common gene in ARNSHL in UAE population (Tlili et al., 2017, under revision) and found no mutations. As next-generation sequencing-based methods (WES and specially the gene panel test) became a preferable strategies to screen mutations in several genes at the same time, taking into consideration their cost effectiveness and reduced time consumption as compared to other genetic testing methods [2], we performed WES analysis on the proband and we were able by Sanger sequencing to identify the causative mutation in the *ILDR1* gene. In 2011, this gene has been implicated in ARNSHL in 11 families linked to *DFNB42* locus [10]. So far, several mutations in the *ILDR1* gene have been described [2,3,11,12,19,20].

The longest isoform of the *ILDR1* gene produces a transmembrane protein with 546 amino acids. It contains a signal peptide, an extracellular immunoglobulin (Ig) superfamily domain, a transmembrane domain, a cysteine-rich and an arginine-rich domain, an LSR (lipolysis stimulated lipoprotein receptor) domain, a dileucine motif, and a 14-3-3 binding site [8,10]. The c.804delG (p.Glu269ArgfsTer4) mutation is predicted to produce a mutant protein lacking the arginine-rich domain and the 14-3-3 binding site. These two regions are very conserved among several species, which suggests an important biological function. In fact, arginine-rich motifs are found in important regulatory complexes, and they are predicted to mediate protein-protein interaction [21] or to bind a functional domain of a protein to an RNA [22,23]. The 14-3-3 binding sites are also important since 14-3-3 domain bind target proteins and modulate their activity, stability and subcellular localization. Furthermore, 14-3-3 domain contributes to protein complex formation [24]. In 2015, Higashi et al., documented that hairs cells in the *Ildr1* knockout mice develop normally, but begin to degenerate two weeks after birth. Thus, they suggest that in the absence of ILDR1 gene, hair cells undergo a postnatal degeneration. At P35, all knockout mice had profound sensorineural hearing loss associated with a complete loss of outer hair cells and a disorganization of most stereocilia in inner hair cells [25].

The phenotype of the UAE family reported here, is similar to some of the previously reported DFNB42 families. Affected members have severe to profound sensorineural hearing loss. A review of all mutations reported up to date, the in-silico analysis of their consequences at the protein level (Table 1 and Fig 3) and the correlation with their associated phenotype (Table 2), suggested that the mutation in the extracellular domain, induces moderate deafness that is detected at low frequencies, but any mutation disturbing the intracellular domain of the *ILDR1* protein will result in a severe deafness at low frequencies. An exception to our finding are p.Val102Glu and p.S406X mutations described by Mehrjoo et al., 2015 (Table 2). Hence, we suggest for DFNB 42 linked families with moderate hearing loss at low frequencies to screen exons 1, 2 and 3 as they encode the extracellular domain of *ILDR1* gene.

**Table 2:** Mutations in *ILDR1* gene and associated phenotypes.

Mutation (cDNA)	Mutation (protein)	Affected Domain (s)	Human phenotype
c.3G>A	p.Met1Ile*ext*+136	Signal peptide and extracellular domain	Moderate to profound
c.59-5_88del	p.Gly20_Thr31del	Signal peptide and extracellular domain	Moderate to profound
c.82delG	p.V28SfsX31	Extracellular, transmembrane and intracellular domains	N/A
c.206C>A	p.Pro69His	Extracellular domain	Post-lingual onset and partial deafness
c.290 G>A	p.Arg97Gln	Extracellular domain	N/A
c.305T>A	p.Val102Glu	Extracellular domain	Severe to profound
c.325_333dupAATGAGCCC	p.Asn109_Pro111dup	Extracellular domain	Moderate to profound
c.411delG	p.Trp137CysfsX25	Extracellular domain	N/A
c.499+1G>A	p.Trp168LysfsTer47	Transmembrane and intracellular domains	Severe
c.583C>T	p.Gln195X	Intracellular domain	Severe to profound
c.804del G	p.Glu269ArgfsTer4	Intracellular domain	Severe to profound
c.820C>T	p.Q274X	Intracellular domain	N/A
c.942C>A	p.C314X	Intracellular domain	N/A
c.1032delG	p.Thr345ProfsX20	Intracellular domain	Severe
c.1135G>T	p.Glu379X	Intracellular domain	Severe to profound
c.1180delG	p.Glu394SerfsX15	Intracellular domain	Severe
c.1217-1218delTC	p.S406X	Intracellular domain	Moderate to profound
c.1358G>A	p.Arg453Gln	Intracellular domain	Severe to profound

N/A: not available

As low frequencies reflect the activity of the apical part of the cochlea, we suggest that the extracellular domain of *ILDR1* protein is not very crucial in the apical part of the organ of Corti. However, any alteration of the intracellular domain affects the totality of this organ and results in a severe to profound hearing loss phenotype. This hypothesis is supported by *ILDR1* knockout mice results. [25–27]. In fact, all knockout mice unable to express ILDR1 intracellular domain showed severe to profound deafness. Furthermore, scanning electronic microscopy and/or immunocytochemistry analysis in adult mutants, revealed that all outer and inner hear cells were degenerated in the apical, middle and basal turns of the cochlea [25–27]. Moreover, differential gene expression profiles along the axis of the mouse cochlea have been established [28,29]. As an example, the *Tectb* gene, responsible for low frequency hearing loss in mouse [30], is 23-fold more expressed in the apical turn, which is sensitive to low frequencies, compared to middle and basal turns [29]. To explain the phenotypic variability related to *ILDR1* mutations, we suggest a mechanism in which a differential protein functional profile along the axis of the cochlea occurs; in the middle and basal turns of the cochlea, both extracellular and cytoplasmic domains are essential for the structural integrity and functionality of inner ear tricellular tight junctions, however in the apical turn, only the cytoplasmic domain is required.

In addition to the previous phenotype-genotype correlation based on the position of the mutation, we can also consider the nature of the mutation as a determinant factor for the phenotypic variability of ILDR1 mutants. In fact, most of mutations associated with severe to profound hearing loss (except p.Val102Glu and p.Arg453Gln) are nonsense mutations that likely lead to nonsense mediated decay of the mutated mRNA and prevent translation of ILDR1 protein. On the other hand, non-truncated mutations associated with moderate to profound hearing defect (except p.S406X) seem more often to allow production of the mutated protein. Based on these observations, we can suggest that the presence of the mutated ILDR1 protein in the cochlea results in a moderate to profound hearing loss. However, the absence of the ILDR1 protein results in a severe to profound phenotype. This gradient of hearing impairment related to inactivating variants (stop mutations or frame shifts) and non-inactivating variants (missense mutations) has been reported in several studies. As example, Cryns et al, found in 2004 that 35delG homozygotes (an inactivating mutation) have significantly more hearing impairment, compared with 35delG/non-35delG compound heterozygotes. They also showed that people with two non-35delG mutations have even less hearing impairment [31].

## Conclusion

To conclude, this report provides a review of all *ILDR1* mutations with phenotype genotype correlation alongside the molecular diagnosis of a consanguineous UAE family with five deaf individuals found to be homozygous for a novel frameshift mutation c.804delG.

## Supporting information

S1 Table Clinical assessment of affected individuals. (DOCX)
