# Supplementary material for: Identification of a novel frameshift mutation in the ILDR1 gene in a UAE family, mutations review and phenotype genotype correlation
Source: PLoS One. 2017 Sep 25;12(9):e0185281. doi: 10.1371/journal.pone.0185281 (PMC5612695; doi:10.1371/journal.pone.0185281)
Supplement: S1 Table — (DOCX) [file pone.0185281.s001.docx]

| # | Age | Age at onset | Tympanometry | Types of hearing loss | Degree of hearing loss | Other complications |
| --- | --- | --- | --- | --- | --- | --- |
| II-1 | 40 | Congenital | Normal | Sensorineural | Severe to profound | No |
| II-4 | 38 | Congenital | Normal | Sensorineural | Severe to profound | Allergic asthma after puberty |
| II-6 | 37 | Congenital | Normal | Sensorineural | Severe to profound | No |
| II-7 | 36 | Congenital | Normal | Sensorineural | Severe to profound | Allergic asthma since child |
| II-11 | 28 | Congenital | Normal | Sensorineural | Severe to profound | No |
